# ^18^F-Sodium Fluoride PET as a Diagnostic Modality for Metabolic, Autoimmune, and Osteogenic Bone Disorders: Cellular Mechanisms and Clinical Applications

**DOI:** 10.3390/ijms22126504

**Published:** 2021-06-17

**Authors:** Peter Sang Uk Park, William Y. Raynor, Yusha Sun, Thomas J. Werner, Chamith S. Rajapakse, Abass Alavi

**Affiliations:** 1 Department of Radiology, Hospital of the University of Pennsylvania, 3400 Spruce Street, Philadelphia, PA 19104, USA; peter.park@pennmedicine.upenn.edu (P.S.U.P.); william.raynor@pennmedicine.upenn.edu (W.Y.R.); tom.werner@pennmedicine.upenn.edu (T.J.W.); chamith@pennmedicine.upenn.edu (C.S.R.); 2 Perelman School of Medicine at the University of Pennsylvania, 3400 Civic Center Boulevard, Philadelphia, PA 19104, USA; yusha.sun@pennmedicine.upenn.edu; 3 Department of Orthopaedic Surgery, Hospital of the University of Pennsylvania, 3400 Spruce Street, Philadelphia, PA 19104, USA

**Keywords:** ^18^F-sodium fluoride, ^18^F-NaF, PET, osteoporosis, Paget’s disease, hyperparathyroidism, ankylosing spondylitis, rheumatoid arthritis, osteosarcoma

## Abstract

In a healthy body, homeostatic actions of osteoclasts and osteoblasts maintain the integrity of the skeletal system. When cellular activities of osteoclasts and osteoblasts become abnormal, pathological bone conditions, such as osteoporosis, can occur. Traditional imaging modalities, such as radiographs, are insensitive to the early cellular changes that precede gross pathological findings, often leading to delayed disease diagnoses and suboptimal therapeutic strategies. ^18^F-sodium fluoride (^18^F-NaF)-positron emission tomography (PET) is an emerging imaging modality with the potential for early diagnosis and monitoring of bone diseases through the detection of subtle metabolic changes. Specifically, the dissociated ^18^F^-^ is incorporated into hydroxyapatite, and its uptake reflects osteoblastic activity and bone perfusion, allowing for the quantification of bone turnover. While ^18^F-NaF-PET has traditionally been used to detect metastatic bone disease, recent literature corroborates the use of ^18^F-NaF-PET in benign osseous conditions as well. In this review, we discuss the cellular mechanisms of ^18^F-NaF-PET and examine recent findings on its clinical application in diverse metabolic, autoimmune, and osteogenic bone disorders.

## 1. Introduction

Bone is a dynamic tissue that is constantly remodeled by the actions of bone-resorbing osteoclasts and bone-forming osteoblasts. Formation of new bone by osteoblasts follows the resorption of older bone by osteoclasts in a process called bone turnover [1]. Osteoclasts are the primary bone-resorbing cells of the body from the hemopoietic stem cell lineage, degrading the bone by attaching itself to the bone matrix, establishing an acidic microenvironment called the sealing zone, and secreting various proteases, such as cathepsin K, that degrade matrix proteins [2]. Meanwhile, osteoblasts are primary bone-forming cells of mesenchymal stem cell origin. The mature osteoblast, which is characterized by the expression of osteocalcin and alkaline phosphatase, builds bone by depositing a collagen type I-rich matrix that serves as a template for hydroxyapatite mineralization [3].

Abnormal bone formation and degradation are the underlying mechanisms behind many pathological bone conditions. When bone resorption becomes excessive as a result of physiological aging or hormonal imbalances, metabolic bone disease, such as osteoporosis, can occur [4]. On the other hand, overactivated osteogenic cells from genetic mutations or cancer, such as osteosarcoma, can ossify tissues outside of the normal skeletal system [5]. Autoimmune conditions can involve both excessive resorption and formation, which can result in spine fusion often seen in ankylosing spondylitis [6]. Regardless of the mechanism, symptoms and pathological manifestations of these conditions are often preceded by molecular alterations not adequately measured by traditional imaging techniques, such as radiographs or dual-energy X-ray absorptiometry (DXA) [7]. Therefore, development of imaging modalities sensitive to these microscopic changes could potentially revolutionize the early clinical management of conditions involving abnormal bone metabolism.

^18^F-Sodium fluoride (^18^F-NaF)-positron emission tomography (PET)/computed tomography (CT) is an emerging imaging modality with great promise for the early diagnosis, treatment, and monitoring of bone disorders. ^18^F-NaF labeled with fluorine-18 is a radioactive tracer that specifically reflects blood flow to the bone and osteoblastic activity in either osseous or soft tissue [8]. Historically, technetium-99m (^99m^Tc)-labeled phosphate-based bone tracers were widely adopted instead of ^18^F-NaF for skeletal imaging and scintigraphy because they were better optimized for gamma cameras and had longer half-lives that allowed for easier storage and delivery. However, ^18^F-NaF has experienced a renaissance as a result of advancements in modern PET scanners that better capture its incidence photons, the wide availability of PET/CT systems from the popularity of ^18^F-fluorodeoxyglucose (^18^F-FDG) in oncological practices, a shortage of ^99m^Tc, and the development of an efficient method of production involving a single-step reaction in a cyclotron [9,10]. Furthermore, ^18^F-NaF offers several advantages over ^99m^Tc-labeled agents. With comparable radiation exposure, ^18^F-NaF has lower binding to protein and has rapid uptake and clearance in the plasma that allows the acquisition of images with low background-to-bone ratios obtainable within 60 to 90 min after tracer administration [11]. Often, whole-body ^18^F-NaF-PET scans are acquired, allowing the detection of ^18^F-NaF-avid lesions throughout the body [12]. The time between ^18^F-NaF administration and PET acquisition has been shown to be a negligent factor in analyzing vascular ^18^F-NaF uptake [13]. Combining ^18^F-NaF-PET with CT has the advantage of providing a means of attenuation correction and anatomical correlation, thereby increasing the sensitivity and specificity in the diagnosis of skeletal disorders and osseous lesions. Meanwhile, combining ^18^F-NaF-PET with MRI allows the simultaneous detection of sites with abnormal ^18^F-NaF uptake and structural changes involving cartilage, bone marrow, and soft tissue, such as inflammation and bone marrow edema [14].

One of the main methods of quantifying ^18^F-NaF uptake and bone turnover with ^18^F-NaF-PET/CT is calculating the standardized uptake value (SUV), which reflects ^18^F-NaF concentration (kBq/mL) in a particular region of interest (ROI) at a single static scan normalized by body weight (kg) and injection activity (MBq) [15]. Another method described by Hawkins et al. measures plasma clearance of ^18^F-NaF to bone mineral expressed as *Ki*, in which the arterial input function is calculated using a 60-min dynamic scan and arterial or venous blood sampling [16]. Specifically, the Hawkins method uses a nonlinear regression method composed of three compartments including plasma, bone extracellular fluid, and bone mineral and four various rate constants describing the movement of ^18^F-NaF through the compartments to calculate the net uptake or clearance of ^18^F-NaF from plasma to the bone. However, due to the difficulty of 60-min imaging and the invasiveness of blood sampling, calculating uptake rather than plasma clearance is preferable in clinical settings [17].

**Figure 1:**
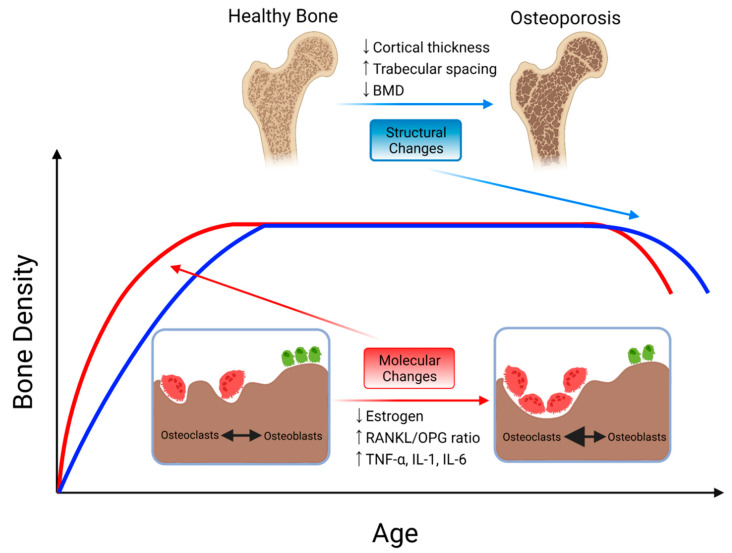
Schematic graph and representation of changes in bone disease with aging in osteoporosis. Molecular changes that favor bone resorption over bone formation such as decreased estrogen, increased RANKL/OPG ratio, and increased inflammatory cytokines such as TNF-α, IL-1, and IL-6 precede structural changes in the bone. Structural changes associated with osteoporotic bone include decreased cortical thickness, increased trabecular spacing, and decreased bone mass density.

^18^F-NaF-PET has been primarily used in the context of metastatic bone diseases, such as prostate cancer; however, this effort has been misguided by a focus on imaging the osseous reaction to skeletal metastases rather than on imaging the cancer cells themselves, which can be accomplished with tumor-specific PET tracers. The specificity of ^18^F-NaF-PET for osteoblastic activity and bone perfusion makes it suitable and ideal for diagnosing and monitoring diverse pathological osseous conditions with abnormal bone turnover and osteoblastic activity (Figure 1) [18,19,20]. In this review, we explore the molecular and cellular basis of ^18^F-NaF-PET for detecting site-specific bone turnover and examine the recent application of ^18^F-NaF-PET as a diagnostic modality for pathological osseous conditions, including metabolic, autoimmune, and osteogenic bone disorders.

## 2. Cellular Basis of Detecting Altered Bone Lesions Using ^18^F-NaF-PET

### 2.1. Osteoblastic Activity

Pathologically altered osteoblastic activity is most straightforwardly captured by ^18^F-NaF-PET, which detects incident photons resulting from positron emission of radioactive fluoride ions that have become incorporated into the hydroxyapatite surface of newly formed bone [21]. Osteoblasts are the main bone-building cells arising from the mesenchymal stem cell lineages. Proliferation and differentiation of osteoblast precursors are maintained by the expression of Runx2, which drives their commitment to the osteoblast lineage by upregulating canonical pathways, such as Hedgehog and Wnt. Subsequent expression of Osterix/SP7 drives the differentiation of mature osteoblasts, which expresses osteocalcin and alkaline phosphatases that can serve as biomarkers for systemic bone turnover activity [22,23]. Excessive osteoblastic differentiation and activity can lead to abnormal bone turnover seen in Paget’s disease or osteosarcoma, which can be assessed by ^18^F-NaF-PET.

### 2.2. Osteoclast–Osteoblast Coupling

Although ^18^F-NaF-PET is specific to osteoblast activity, it can also be sensitive for lytic bone lesions that are accompanied by a component of abnormal osteoblast activity [11]. It is well known that the activities of osteoclasts and osteoblasts are closely coupled to one another—osteoclasts and osteoblasts communicate and interact with each other via cell-to-cell contact and the secretion of cytokines. Osteoclasts, the multinucleated bone-resorbing cells of the body, originate from the myeloid lineage of the hematopoietic stem cells in the marrow. Proliferation and differentiation of osteoclast precursor cells are regulated by macrophage colony-stimulating factor (M-CSF) and receptor activator of nuclear factor-κ B ligand (RANKL), respectively [24,25]. Under pathological conditions, osteoclasts can also become activated by various inflammatory cytokines, such as tumor necrosis factor-α (TNF-α), interleukin (IL)-1, and IL-6 [24]. Osteoblasts directly promote osteoclast differentiation by secreting M-CSF and RANKL as well as their inhibition by secreting osteoprotegerin (OPG), which is a decoy receptor for RANKL. Similarly, osteoclasts can promote osteoblasts and bone formation by releasing transforming growth factor beta (TGF-β) and insulin-like growth factor 1 (IGF-1) from the bone matrix [26,27]. Anti-resorptive therapies, such as denosumab, can obstruct the osteoclast–osteoblast communication by preventing RANKL from binding to receptor activator of nuclear factor-κ B (RANK) on the osteoclast surface, inhibit osteoclast formation, and decrease osteoclast-derived coupling factors that stimulate bone formation by osteoblasts, which can all be detected and monitored using ^18^F-NaF-PET [28,29].

### 2.3. Bone Perfusion

**Figure 2:**
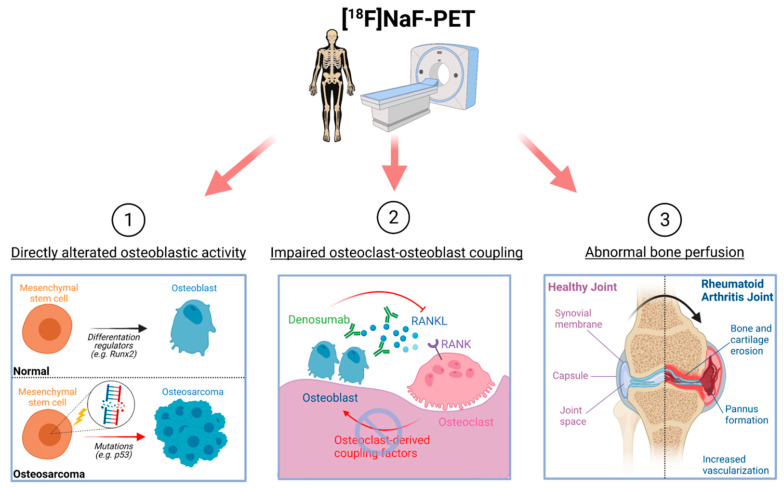
Cellular mechanisms that influence observed ^18^F-NaF activity. ^18^F-NaF-PET depicts bone turnover by detecting direct alterations in osteoblasts, impaired osteoblast–osteoclast coupling, and abnormal bone perfusion.

As ^18^F-NaF is administered intravenously and travels to the region of uptake via blood vessels, differential vasculature to the bone also influences ^18^F-NaF uptake [15]. Variations in regional bone perfusion to the different bones of the body have been previously demonstrated with ^18^F-NaF-PET, and abnormal ^18^F-NaF uptake in pathological conditions may reflect altered vascularity and angiogenesis, which are known to be associated with bone turnover [30]. In fact, angiogenesis is implicated in several osteogenesis processes, such as bone development, fracture repair, and pannus formation in rheumatoid arthritis (RA). Angiogenesis is stimulated under hypoxic conditions by hypoxia-inducible factors (HIFs), leading to the expression of the master transcriptional regulator vascular endothelial growth factor (VEGF). In normal bone development, VEGF couples angiogenesis and osteogenesis together, regulating the proliferation of endothelial cells and stimulating osteogenesis [31]. In pathological conditions, such as RA, pannus formation in the joint is characterized by increased vascularity, while osteoporotic bone is hypothesized to exhibit decreased bone perfusion [32,33]. As such, ^18^F-NaF-PET may be a suitable modality for examining abnormal blood flow to the bone in many disease conditions. Overall, elucidating the cellular mechanisms behind pathological ^18^F-NaF uptake will widen the clinical application of ^18^F-NaF-PET and strengthen the rationale and molecular basis for its use (Figure 2).

## 3. ^18^F-NaF-PET in Metabolic Bone Disorders

### 3.1. Osteoporosis

Osteoporosis is a metabolic bone disorder characterized by abnormally low bone mineral density (BMD) and impaired bone microstructural integrity, leading to fragile bone greatly susceptible to fractures and ultimately decreased quality of life. Molecularly, osteoporosis is known to result from an imbalance in bone homeostasis in which bone resorption by osteoclasts disproportionally exceeds bone formation by osteoblasts as well as decreased BMD from impaired bone perfusion associated with aging [4,34,35].

The primary demographic group affected by osteoporosis includes postmenopausal women with excessive bone degradation associated with increased aging and estrogen deficiency, which increase osteoclast activity [36]. Vertebral and hip fractures are the most common clinical manifestations of osteoporosis, often resulting in complications, such as pain, decreased mobility, disability, and mortality [37]. A clinical diagnosis of osteoporosis is made based on either fragility fracture or quantitative measurement of BMD using dual-energy X-ray absorptiometry (DXA) [38]. While DXA remains the most frequently used modality to measure BMD and diagnose osteoporosis, it has several downsides. These limitations include low resolution to bone microarchitecture and quality, lack of three-dimensional information, and inability to discriminate between cortical and trabecular bone [7,39]. Since molecular alterations in the bone often precede gross structural changes seen in osteoporosis, use of modalities with greater resolution, depth, and molecular sensitivity, such as ^18^F-NaF-PET/CT, could be revolutionary for early diagnosis of osteoporosis and other metabolic bone diseases. 

**Figure 3:**
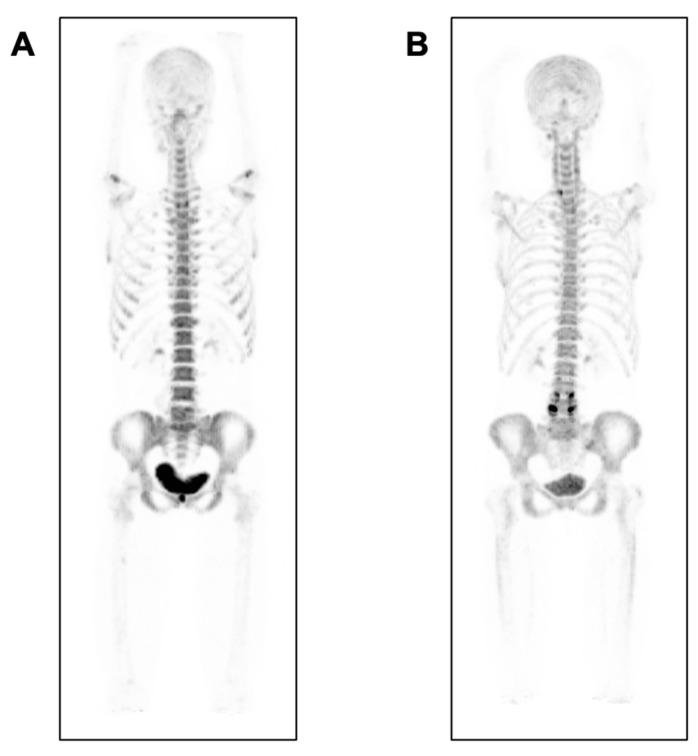
Aging and ^18^F-NaF uptake in the bone. Maximum intensity projection ^18^F-NaF-PET images of two healthy subjects, (**A**) a 26-year-old female and (**B**) a 62-year-old female. The difference in ^18^F-NaF uptake is visible particularly in the spine, pelvis, and proximal femur, which can be quantified in longitudinal studies to monitor disease progression and therapeutic response.

^18^F-NaF-PET is a sensitive modality capable of monitoring the molecular effects of osteoporosis (Figure 3). A study with 72 postmenopausal women who were placed into normal, osteopenic, or osteoporotic groups according to their BMD T-score discovered that the osteoporotic group had significantly decreased plasma clearance of ^18^F-NaF to the bone mineral compartment of the lumbar spine compared to both osteopenic and normal groups, suggesting that ^18^F-NaF-PET is a useful biomarker capable of detecting the summative effect of impaired osteoblast function and decreased bone perfusion in patients with osteoporosis [40]. Another study used ^18^F-NaF-PET/CT-derived SUV measurements to derive a score called the bone metabolism score (BMS) that could serve as a biomarker of age-related metabolic changes at the femoral neck, which is one of the most common sites for osteoporotic bone fracture. These studies indicate that ^18^F-NaF-PET/CT could be clinically implemented to determine osteoporotic changes in the bone with age and be used for better guided therapeutic decision-making [41].

In addition, ^18^F-NaF-PET has been shown to be capable of detecting molecular alterations due to therapeutic interventions in patients with osteoporosis, possibly earlier than other traditional markers or imaging modalities. A study of 24 postmenopausal women with history of glucocorticoid-induced osteoporosis employed ^18^F-NaF-PET to determine if there were any significant changes in the ^18^F-NaF uptake in the bones upon treatment with alendronate. Alendronate is an anti-resorptive agent and a type of bisphosphonate, which binds to hydroxyapatite bone, induces osteoclast apoptosis, and decreases bone resorption [42]. After 3 months of treatment, a significant decrease in ^18^F-NaF uptake in the lumbar spine was detected, while there were no significant changes in BMD or serum bone-specific alkaline phosphate (BSALP) levels in the same timeframe [43]. Decreased ^18^F-NaF uptake can be explained by the observation that bisphosphonates can indirectly inhibit bone formation by directly inhibiting bone resorption, as the two processes are closely coupled [44]. Increased BMD from the bisphosphonate treatment reflects the relatively slower attenuation of osteoblast activity compared to that of osteoclasts, resulting in net bone formation [39]. Similarly, a study of 18 women considered osteoporotic or osteopenic by their BMD T-scores revealed that after six months of bisphosphonate risedronate therapy, significant decreases in mean vertebral *Ki* and BSALP levels were detected [45].

Overall, studies suggest that ^18^F-NaF-PET may be used to detect decreased bone turnover activity during anti-resorptive therapy. In addition to detecting the effects of bisphosphonates, ^18^F-NaF-PET has also been used to monitor the effect of bone anabolic agents, such as teriparatide, which increases both osteoblast and osteoclast activities. Teriparatide treatment has been observed to increase ^18^F-NaF uptake in the bones of osteoporotic patients [46,47]. It would be interesting to observe the effect of other therapeutic anti-resorptive agents, such as denosumab, on the ^18^F-NaF uptake of osteoporotic patients. It has been shown that denosumab treatment in patients with fibrous dysplasia/McCune–Albright syndrome, a rare condition in which fibrous tissues replace the bone, decreases the disease burden measured by ^18^F-NaF-PET/CT, as well as serum procollagen-1 N-terminal peptide (P1NP) and alkaline phosphatase levels [29].

Further investigation on the effects of osteoporosis and its treatments in different skeletal sites could be crucial for the implementation of ^18^F-NaF-PET into the clinical management regimen, given that ^18^F-NaF uptake may vary depending on the type and site of the bone being analyzed as well as the type of bisphosphonate used. A study using ^18^F-NaF-PET to examine bone metabolism in the lumbar spine, hip, and femur after discontinuation of either alendronate or risedronate in 20 postmenopausal women found that bone metabolism at spine and femoral neck did not significantly change after discontinuation for both alendronate and risedronate groups, but ^18^F-NaF uptake measured by SUV significantly increased at femoral shaft and hip in the alendronate treatment group only [48]. Differential ^18^F-NaF uptake depending on bone has also been demonstrated previously. For instance, correlations between ^18^F-NaF uptake measured by SUV and age at the humeral shaft and lumbar spine have been reported to show opposite trends [49]. Overall, the validation of ^18^F-NaF-PET for the treatment and diagnosis of osteoporosis with further studies will certainly enhance the way metabolic bone diseases are diagnosed and treated [7,50]. 

### 3.2. Paget’s Disease

Paget’s disease of the bone, or osteitis deformans, is a metabolic bone disease caused by increased bone turnover. While it has traditionally been classified as a disease of osteoclasts, abnormality of osteoblasts in the condition is also recently being recognized. Pathogenesis of Paget’s disease is characterized by increased activation of multinucleated osteoclasts and excessive bone resorption followed by the abnormal bone formation of osteoblast that results in structurally unstable and disorganized new bone [51]. The accelerated bone remodeling and turnover cause overgrowth of bone at either single (monostotic) or multiple (polyostotic) sites in the body, such as the skull and spine. Bisphosphonates remain the first-line of treatment, as they interfere with osteoclast function and survival to decrease the rate of bone turnover [52]. 

^18^F-NaF-PET has great potential to detect and monitor Paget’s disease, even when it remains in asymptomatic form, because ^18^F-NaF-PET is sensitive to the increased osteoblast activity seen in pagetic bone. A study of 7 patients with Paget’s disease involving the vertebrae demonstrated increased ^18^F-NaF plasma clearance in pagetic vertebra as measured by *Ki* compared to unaffected bone, suggesting that ^18^F-NaF-PET is a suitable modality for both detecting and localizing pagetic bone [53]. ^18^F-NaF-PET has also been used to monitor and quantify treatment progress in Paget’s disease as well. In a study with 14 patients with either monostotic or polyostotic forms of Paget’s disease, ^18^F-NaF-PET detected significantly decreased ^18^F-NaF uptake as measured by the maximum SUV after one month of bisphosphonate treatment in all but one patient. While ^18^F-NaF uptake remained at high levels at one month compared to normal control bone, all biochemical markers of bone turnover were normal in six of nine patients with monostotic disease, suggesting that ^18^F-NaF-PET may be a more sensitive method of monitoring therapy response, especially for subjects with the monostotic form [54]. 

### 3.3. Hyperparathyroidism

Hyperparathyroidism is an endocrine condition with excess production of parathyroid hormone (PTH) by parathyroid glands and hypercalcemia. Routine measurements of serum calcium levels allow for early detection of hyperparathyroidism, but patients with asymptomatic hyperparathyroidism can still present with osteoporosis and vertebral fractures as a result of abnormal bone homeostasis [55]. PTH activates osteoclasts indirectly by inducing osteoblasts to express RANKL and increase calcium levels by stimulating bone resorption [56]. Rarely, severe forms of primary, secondary, and tertiary hyperparathyroidism can manifest as osteitis fibrosa cystica, which are fibrous cysts resulting from excessive bone destruction by osteoclasts. When the cysts are filled with brown hemosiderin deposits, a non-neoplastic lesion called brown tumor can arise [57]. 

^18^F-NaF-PET/CT is useful for detecting osteitis fibrosa cystica and brown tumors by showing either increased osteoblast activity or high vascularization in the lesions [58]. There has been a case report of a 16-year-old boy with osteitis fibrosa cystica shown to have increased tracer avidity on ^18^F-NaF-PET/CT [59]. A retrospective series of eight patients with primary hyperparathyroidism employed ^18^F-NaF-PET/CT to identify a total of 72 brown tumors with an average maximum SUV of 17.5 ± 7.8 [60]. In another case report, a patient without any bone or joint pain was diagnosed with secondary hyperparathyroidism by the detection of a brown tumor while ruling out any bone malignancy with ^18^F-NaF-PET/CT, highlighting the sensitivity of this imaging modality [61]. 

## 4. ^18^F-NaF-PET in Autoimmune Diseases

### 4.1. Ankylosing Spondylitis

Ankylosing spondylitis (AS) is a rheumatic disease characterized by inflammatory arthritis of joints in axial bones, such as those of the spine as well as the sacroiliac joints. It commonly manifests as chronic back pain, predominantly affecting males under the age of 45 years. Patients with severe AS can suffer from fusion (ankylosing) of the spine as well as syndesmophyte formation due to abnormal bone deposition in the context of a chronic inflammatory state [6]. The main imaging modalities currently used to diagnose AS remain conventional radiography and magnetic resonance imaging (MRI). Radiographs are used to identify abnormalities in the sacroiliac joint such as syndesmophytes, erosions, and sclerosis, but they are often subtle and not identified until the advanced stages of the disease. On the other hand, MRI has the advantage of detecting inflammatory changes with greater sensitivity at earlier disease progression [62]. Regardless, both modalities remain insensitive to the pathological bone turnover present in AS, and there is a dire need to improve methods of early detection and characterization.

Integrating MRI with ^18^F-NaF-PET may enhance the early diagnoses of AS by the detection of inflammatory lesions with abnormal bone turnover and may be useful in investigating the relationship between molecular and structural abnormalities seen in AS. A study investigating 12 male patients with AS discovered that anterior vertebral corners with spinal inflammation detected by MRI and syndesmophyte identified by conventional radiograph had significantly higher ^18^F-NaF uptake measured by maximum SUV compared to control regions, suggesting a link between inflammation and osteoblastic activity [63]. A follow-up of the study after two years found that new syndesmophytes formed significantly more frequently in anterior vertebral corners with increased ^18^F-NaF uptake lesions at baseline compared to those without, suggesting that increased ^18^F-NaF uptake may predict new syndesmophyte formation in the later disease stages. On the other hand, neither acute nor chronic inflammatory lesions assessed by MRI were predictive of future syndesmophyte formation [64]. Additional studies using ^18^F-NaF-PET/MRI have also shown that increased ^18^F-NaF uptake was most associated with areas of acute spinal inflammation marked as bone marrow edema, while pathologic lesions such as erosions, sclerosis, and ankylosis did not [14,65]. 

Another hybrid approach for analyzing abnormal bone-turnover lesions with high spatial resolution and diagnosing AS is ^18^F-NaF-PET/CT. In a single examination, ^18^F-NaF-PET/CT allows the assessment of the entire skeleton and allows quantification of bone turnover. A study of 15 patients with AS used a ratio of SUV in the sacroiliac joint to that of sacrum derived from ^18^F-NaF-PET/CT with a defined cut-off to diagnose sacroiliitis with an overall sensitivity of 80% [66]. A retrospective study of 68 patients with AS similarly validated the diagnostic value of ^18^F-NaF-PET/CT, which had a diagnostic rate of 72.1% with sensitivity to enthesopathy, syndesmophytes and symmetric sacroiliitis [67]. Another study involving 29 patients with AS demonstrated that there was a significant correlation between the number of ^18^F-NaF-PET/CT positive sites and disease activity indexes, suggesting that ^18^F-NaF-PET may be useful for quantifying the severity of the disease [68]. 

^18^F-NaF-PET/CT may serve as a biomarker for predicting treatment response in AS as well. In patients with AS receiving a TNF-α blocker, the maximum SUV of the spine derived from ^18^F-NaF-PET/CT reliably predicted therapy response as measured by the Bath Ankylosing Spondylitis Disease Activity Index (BASDAI) in responders and non-responders [69]. Similarly, a semi-quantitative index called lesion-to-background ratio derived from ^18^F-NaF-PET/CT images of the spine in AS patients using anti-TNF-α or anti-inflammatory drugs had a significant correlation with follow-up BASDAI score, suggesting that ^18^F-NaF-PET/CT could be helpful in predicting treatment response [70].

### 4.2. Rheumatoid Arthritis

Rheumatoid arthritis (RA) is a chronic inflammatory disease of the joint that leads to the destruction and erosion of cartilage and bone. In RA, various immune cells, such as macrophages and T cells, infiltrate the synovial fluid and secrete inflammatory cytokines, such as TNF-α and IL-6, to stimulate bone resorption [71]. Damage to the joints by RA severely limits mobility, increases risks for premature death, and carries socioeconomic costs [72]. ^18^F-NaF-PET/CT has been demonstrated to be sensitive to bone erosions seen in the joints of RA patients. In a study involving 12 RA patients undergoing biological treatment, ^18^F-NaF uptake measured by maximum SUV was significantly higher in RA-affected, erosive joints compared to non-erosive ones [73]. ^18^F-NaF uptake was also found to correlate with the Kellgren–Lawrence scores in nine RA patients who underwent both ^18^F-NaF-PET/CT imaging and conventional radiography of the knee [74]. In addition to identifying areas of erosion due to RA, ^18^F-NaF-PET/CT could be used for the early detection of RA disease activity. In a case report of a 67-year-old male, ^18^F-NaF-PET/CT detected 18 joints with ^18^F-NaF abnormalities that were undetectable by ^18^F-FDG, ultrasound, or clinical examinations prior to RA diagnosis confirmed later [75]. In addition to abnormal bone turnover in the joints, ^18^F-NaF-PET/CT has been shown to detect abdominal aortic calcification in RA patients [76]. 

Inhibitory effects of the RA microenvironment on osteoblasts raise the possibility that increased ^18^F-NaF uptake in RA-affected joints may reflect significantly increased vascularity and ^18^F-NaF flow to the skeletal lesion in addition to increased bone turnover [77,78]. Thus, clarifying the source of increased ^18^F-NaF uptake may be helpful in determining the precise cellular mechanism behind joint erosions in RA.

## 5. ^18^F-NaF-PET in Osteogenic Bone Disorders

### 5.1. Osteosarcoma

Osteosarcoma is a primary malignant bone tumor characterized by excessive production of osteoid and immature bone due to overactive bone-forming malignant cells [79]. The malignant osteoblastic cells arise from the accumulation of gene mutations, such as loss of p53, that results in increased osteogenic differentiation of mesenchymal stem cells [80,81]. There have been numerous case reports identifying osseous lesions and metastases in patients with osteosarcoma using ^18^F-NaF-PET [82,83,84,85,86]. In a prospective evaluation of ^99m^Tc-MDP scintigraphy, ^18^F-NaF-PET/CT, and ^18^F-FDG-PET/CT for identifying skeletal metastases, researchers were able to use ^18^F-NaF-PET/CT to identify skeletal lesions and metastases not identified by the other two modalities, highlighting the sensitivity of ^18^F-NaF-PET/CT for detecting osseous lesions [82]. In a study of patients with osteoblastic skeletal metastatic diseases, such as osteosarcoma, ^18^F-NaF-PET/CT was employed to calculate the skeletal tumor burden using a maximum SUV threshold of 10 in a reproducible way between observers [87]. Measurements derived from ^18^F-NaF-PET/CT may provide patients and clinicians with a quantifiable way of monitoring disease progression and management [87,88]. In fact, in a clinical trial of 18 patients with osteosarcoma receiving one to six cycles of radium-223 dichloride for treatment, researchers used ^18^F-NaF-PET/CT to derive a new measurement called NAFCIST (Na^18^F PET response Criteria in Solid Tumors) that significantly correlated with overall survival rate [89]. As such, ^18^F-NaF-PET/CT is a promising modality to identify and monitor osteosarcoma and osteoblastic metastases in a quantifiable manner.

### 5.2. Melorheostosis

Melorheostosis is a rare, benign bone disorder characterized by bone overgrowth typically in the long bones leading to pain and functional disabilities. While the molecular mechanism behind melorheostosis is still under investigation, it is suspected that enhanced ERK1/2 activation and pathway results in greater osteoblast surface and increased deposition of unmineralized bone matrix or osteoid [90]. The “dripping candle wax” appearance on conventional radiography followed by sclerotic lesions on CT have been traditionally used to confirm the diagnosis. Melorheostotic lesions in either the skeleton or soft tissue exhibit highly increased bone-turnover and ^18^F-NaF uptake, suggesting that ^18^F-NaF-PET/CT could be highly valuable for both diagnoses and monitoring of melorheostosis and its treatment [90,91,92]. A clinical study on melorheostosis employed ^18^F-NaF-PET/CT to confirm its diagnosis and subsequent exclusion of patient subjects if they did not exhibit increased ^18^F-NaF uptake in the lesions [93]. The combined nature of ^18^F-NaF-PET/CT may enable clinicians to more efficiently and comprehensively diagnose and examine melorheostosis compared to other imaging modalities—PET can determine the intensity and the extent of bone overgrowth, while CT can be used to locate and visualize ossifications and tissue/bone abnormalities [94]. Another advantage of ^18^F-NaF-PET/CT may be its ability to detect early-state melorheostotic lesions with low-level density on CT through the recognition of significant ^18^F-NaF regional uptake [95].

### 5.3. Fibrodysplasia Ossificans Progressiva

^18^F-NaF-PET/CT shows great promise for early detection and monitoring of heterotopic ossification, or external growth of bone outside of the normal skeletal system [50,96]. Recently, the potential of ^18^F-NaF-PET/CT in diagnosing and predicting the onset of heterotopic ossification in fibrodysplasia ossificans progressiva (FOP) has been gaining traction. FOP is a rare autosomal dominant genetic disorder characterized by inflammatory episodes or “flare-ups” that often precede endochondral heterotopic ossification, which often leads to pain and immobility. Traditionally, FOP imaging typically involves plain radiographs or CT scans. Plain radiographs are cheap and readily available, but they cannot detect lesions undergoing early inflammatory stage and are limited in calculating the volume of heterotopic bone. CT scans allow volumetric measurements but are also of limited value in detecting subtle edema associated with early stages of flare-ups [97,98]. ^18^F-NaF-PET has been shown to be capable of detecting the flare-ups and predicting the location of heterotopic ossification in early disease progress before it is detectable by CT scan alone [99,100]. In a case study of an FOP patient who underwent maxillofacial surgery, ^18^F-NaF-PET/CT was used to predict the onset of new heterotopic bone in a region with increased ^18^F-NaF uptake [99]. In a different study, increased ^18^F-NaF uptake was associated with six progressive but asymptomatic heterotopic lesions that were found in four out of five FOP patients. These results suggest that ^18^F-NaF-PET is capable of identifying heterotopic ossification lesions without any preceding flare-ups, demonstrating superiority over MRI [101,102]. In fact, ^18^F-NaF is currently the only in vivo biomarker recognized and available for detecting and monitoring the progression of FOP [103].

## 6. Conclusions

^18^F-NaF-PET is a promising imaging modality for the early detection and monitoring of pathological bone diseases that affect osteoblast activity, osteoclast–osteoblast coupling, and bone perfusion. The clinical application of ^18^F-NaF-PET has been examined in a wide variety of metabolic, autoimmune, and osteogenic bone disorders. Studies of metabolic bone diseases, such as osteoporosis, have confirmed that ^18^F-NaF-PET is a suitable imaging modality for detecting abnormal bone homeostasis as well as therapeutic responses to anti-resorptive therapies. In inflammatory joint disorders, such as AS and RA, ^18^F-NaF-PET could be employed to identify future pathological sites involving syndesmophyte formation or bone erosion. Additionally, the use of ^18^F-NaF-PET for the diagnosis of rare osteogenic bone disorders or osteosarcomas with significant bone formation activity is corroborated by the literature. Overall, research on the diagnostic role of ^18^F-NaF-PET is expanding, with the potential for revolutionizing the delivery of patient care.
